# Living in the Leaf Litter: Integrative Taxonomy of the *Trichomycterus nigroauratus* Group, a Catfish Clade from an Endangered Tropical Biodiversity Hotspot Supporting Areas of Endemism for Conservation and Two New Species

**DOI:** 10.3390/biology15201829

**Published:** 2026-10-13

**Authors:** Wilson J. E. M. Costa, Maria Anaïs Barbosa, José Leonardo O. Mattos, Paulo J. Vilardo, Pedro F. Amorim, Marina Petrungaro, Axel M. Katz

**Affiliations:** Laboratory of Systematics and Evolution of Teleost Fishes, Institute of Biology, Federal University of Rio de Janeiro, Rio de Janeiro CEP 21941-902, Brazil; anaisbarbosasv@gmail.com (M.A.B.); jlomattos@gmail.com (J.L.O.M.); kpaulojose@gmail.com (P.J.V.); pedro_f_a@hotmail.com (P.F.A.); marinapetrungaro17@gmail.com (M.P.); axelmk@gmail.com (A.M.K.)

**Keywords:** Atlantic Forest, conservation status, species delimitation

## Abstract

Mountain ranges of southeastern Brazil shelter a great diversity of endemic species, making the local Atlantic Forest among the most important and endangered biodiversity hotspots in the world, but some fish groups are still little known. The primary focus of the current study is the *Trichomycterus nigroauratus* group, a catfish clade with species living associated with leaf litter, endemic to the upper and middle Rio Paraiba do Sul basin, with all known species described after 2008. We performed an integrative taxonomic approach combining molecular species delimitation analyses and morphology, which inferred a total of six species, two of which are new and here described, in six disjunct areas. Five areas are coincident with distribution areas of other 11 congeners, supporting areas of endemism. We assessed the conservation status by calculating the extent of occurrence and area of occupancy for each of the 16 species occurring in the five areas of endemism. The Serra da Mantiqueira was considered a biodiversity hotspot for trichomycterine catfishes. The approach followed in this study, combining Integrative Taxonomy to delimit areas of endemism with criteria for assessing conservation status, holds great potential as a method for selecting priority areas for biodiversity conservation.

## Simple Summary

This study focuses on a group of small catfishes, the *Trichomycterus nigroauratus* group, found exclusively in the mountainous areas of the Rio Paraíba do Sul basin in southeastern Brazil. This basin harbours the greatest diversity of *Trichomycterus* catfishes within the Atlantic Forest biome, a high biodiversity region threatened by environmental degradation. These catfishes inhabit leaf litter on stream bottoms and remain poorly understood, with some populations lacking precise identification. To address this problem, we conducted an analysis combining molecular and morphological data, leading us to recognize a total of six species, two of which were previously unknown to science and are described here. The distribution ranges of five of these species overlap with the distribution of other species of the same genus but belonging to different groups. Two neighbouring areas in the Serra da Mantiqueira harbour the highest concentration of species, two of which are classified as endangered based on our assessment, making this region a biodiversity hotspot for trichomycterine catfishes and, consequently, a priority area for conservation.

## 1. Introduction

The Atlantic Forest of eastern South America is among the most diverse and most threatened biodiversity hotspots in the world [1]. Much of the Atlantic Forest biodiversity is concentrated in mountain ranges of southeastern Brazil, such as the Serra do Mar and Serra da Mantiqueira, both containing many endemic species [1,2]. Consequently, important protected areas for biodiversity conservation are situated in areas within these mountain ranges (e.g., Parque Nacional do Itatiaia and Parque Nacional da Serra dos Órgãos). However, the delimitation of biodiversity hotspots and priority areas for conservation is generally based on data on the distribution of terrestrial endemic flora and fauna [1], which do not necessarily correspond to areas of endemism of aquatic organisms.

The Rio Paraíba do Sul basin (hereafter RPSB), a river basin that has suffered severe environmental degradation in recent centuries due to its location between the largest urban and industrial centres of the country (e.g., [3,4]), drains the Serra da Mantiqueira and Serra do Mar simultaneously. Among river basins of eastern Brazil, RPSB shelters the highest species diversity of *Trichomycterus* Valenciennes, 1832 (Siluriformes: Trichomycteridae), a genus of catfishes typically inhabiting fast-flowing streams of mountain ranges of eastern South America [5]. A total of 22 species of *Trichomycterus* belonging to different species groups of five subgenera are endemic to RPSB [6,7]. These species are not homogeneously distributed throughout the basin, but rather they have their distributions mostly concentrated in certain mountainous areas, suggesting the occurrence of separate areas of endemism associated with the mountain environment [6,8].

Four species of *Trichomycterus* endemic to RPSB belong to the *Trichomycterus nigroauratus* group (hereafter TNG) of the subgenus *Trichomycterus* [5], the less studied group in this region. TNG is endemic to drainages of the middle and upper sections of RPSB and comprises *Trichomycterus maculosus* Barbosa & Costa, 2010, *Trichomycterus mutabilicolor* Costa, 2022, *Trichomycterus nigroauratus* Barbosa & Costa, 2008 and *Trichomycterus quintus* Costa, 2020 [8]. TNG is diagnosed by included species having an elongate, narrow and straight posterior process in the pelvic bone (vs. relatively broader and curved when present) and is distinguished from all other taxa of the subgenus *Trichomycterus* by having eight pectoral-fin rays (vs. nine) and the posterior margin of the caudal fin straight to slightly convex (vs. concave at least in juveniles) [5]. In addition, TNG species may be unambiguously distinguished from congeners of other subgenera occurring in RPSB by a combination of the following character states: eight pectoral-fin rays (vs. nine, seven or six rays), eye about equidistant from the snout tip and the posterior margin of opercle (vs. conspicuously nearer snout), jaw teeth always pointed (vs. incisiform at least in larger adults), supraorbital pores always paired (vs. single median supraorbital pore between eyes), and maxillary barbel gradually narrowing between its basal portion and distal extremity (vs. abruptly narrowing after basal portion).

Species of TNG are commonly found buried or swimming over leaf litter deposited on stream bottom areas with intense water flow, often below waterfalls [5]. Probably due to living in such a specific aquatic habitat and consequently being overlooked in ichthyofaunal inventories, species of this group are uncommon in ichthyological collections [9]. Furthermore, most TNG species have abrupt ontogenetic changes in colour patterns, and their colouration may be similar to the colour pattern of some unrelated sympatric species, making TNG species recognition in the field difficult [8,9]. Not surprisingly, the first species of TNG was described only 18 years ago [9], thus contrasting with other groups of *Trichomycterus* from RPSB that have been described since the end of the 19th century and the first decades of the 20th century [10,11,12,13], and included in recent taxonomic revisions [6,7,14,15,16].

Despite recent taxonomic advances [8], the taxonomic status of some geographically disjunct populations of TNG is still undetermined. The primary objective of the present study is to perform an integrative taxonomic approach combining morphology and molecular species delimitation analyses to infer species limits and their respective geographical distributions. Additionally, we delimit areas of endemism based on overlapping distribution patterns of *Trichomycterus* species in RPSB and establish priority areas for conservation based on the number of endemic species and conservation status.

## 2. Materials and Methods

### 2.1. Specimens

Field studies were made with collecting permits provided by ICMBio (Instituto Chico Mendes de Conservação da Biodiversidade; permit numbers: 38553-15, 48432-14) and INEA (Instituto Estadual do Ambiente, Rio de Janeiro; permit number: 036/2023), following field studies protocol approved by CEUA-CCS-UFRJ (Ethics Committee for Animal Use of Federal University of Rio de Janeiro; permit numbers: 065/18 and 084/23), as described in our recent papers (e.g., [6]). Most studied material, including comparative material of TNG species (Appendix A), included specimens already available in the fish collection of the Institute of Biology, Federal University of Rio de Janeiro, Rio de Janeiro (UFRJ). Paratypes of the new species were also deposited in Centro de Ciências Agrárias e Ambientais, Universidade Federal do Maranhão (CICCAA). In the list of specimens, C&S means specimens cleared and stained for osteological examination (see below). Geographical names in lists of specimens follow common names used in Brazil.

### 2.2. Morphology

Terminology and methods for taking and describing morphological data are according to our most recent studies on the genus *Trichomycterus* (e.g., [6,17]), which were mostly based on previous studies, comprising [14,16,18] for morphometric and meristic data, ref. [5,19] for bone terminology, and [17,19,20] for cephalic latero-sensory system terminology. Specimens used in osteological comparisons were prepared following Taylor & Van Dyke [21]. Examined specimens of new species not included in the type series were those with unsatisfactory state of preservation (i.e., not correctly fixed). Osteological characters appearing in species descriptions are directed to structures commonly having informative variability to diagnose congeneric species (i.e., mesethmoidal region, jaw suspensorium and opercular series, and parurohyal).

### 2.3. DNA Extraction, Amplification, and Sequencing

Total genomic DNA was extracted from muscle tissue from the right side of the caudal peduncle using the DNeasy Blood & Tissue Kit (Qiagen, Hilden, Germany), using the manufacturer’s protocol. The quality and integrity of Genomic DNA extraction were verified through agarose gel electrophoresis. The PCR amplifications aimed at a fragment of the mitochondrially encoded gene cytochrome b (*CYTB*). The reactions were performed in a 60 μL total volume, primers Cytb Siluri F (5′-CCA CCG TTG TAA TTC AAC TA-3′) and Cytb Siluri R (5′-GAT TAC AAG ACC GGC GCT TT-3′) [22], with the following reagents concentrations: 5 × Green GoTaq Reaction Buffer (Promega, Madison, WI, USA), 1.5 mM MgCl_2_, 1 µM each primer, 75 ng genomic DNA, 0.2 mM each dNTP, and 1 U GoTaq DNA polymerase (Promega). Thermocycling profile: 95 °C for 4 min; 35 cycles of 94 °C for 40 s, 60 °C for 30 s, and 72 °C for 1.5 min; 72 °C for 4 min. A reaction tube containing only reagents without DNA was used as a negative control to check for contamination. The PCR products were purified using the Wizard SV Gel and PCR Clean-Up System (Promega). Sanger Sequencing reactions were performed by Instituto SENAI de Inovação em Biossintéticos (Rio de Janeiro, Brazil). Sequence editing was performed by examining the chromatograms, where forward and reverse reads were assembled into consensus sequences. Low-quality regions at the ends of the chromatograms were removed based on visual inspection, and the sequences were assessed using MEGA 12 [23]. All generated sequences were translated into amino acid residues to verify the absence of premature stop codons or indels using the MEGA 12 programme.

### 2.4. Phylogenetic Analyses

Terminal taxa comprised 19 specimens of TNG from 14 localities, and three outgroups, including two species of the same subgenus, *Trichomycterus paquequerense* (P. Miranda Ribeiro, 1943) and *Trichomycterus santaeritae* (Eigenmann, 1918), and one species of the subgenus *Paracambeva* Costa, 2021, *Trichomycterus septemradiatus* Katz, Barbosa & Costa, 2013. The analyses included topotypes of all described species. The data matrix comprised a *CYTB* fragment of 1078 bp (Appendix B).

Sequence alignment was conducted using the Clustal W algorithm [24] and inspected for gaps and stop codons in the software MEGA 12; none were found. Substitution saturation was assessed using Xia’s index (Iss) implemented in DAMBE v7 [25], and no evidence of saturation was found. The optimal partition scheme and best-fit models were determined using PartitionFinder 2.1.1 [26], using the Corrected Akaike Information Criterion [27,28]; the resulting partition scheme and best-fit models are presented in Appendix C. The ultrametric tree was calculated using Bayesian Inference (BI) in BEAST 1.10.4 [29], applying the following parameters: an uncorrelated relaxed clock with a lognormal distribution, tree prior set as a Yule speciation process [30], and two independent Markov chain Monte Carlo (MCMC) runs, each with 50 million generations, sampling every 1000 generations. Convergence, stationary phase achievement, effective sample size equal to or above 200, and determination of 25% of the burn-in for the MCMC chains were evaluated using Tracer 1.7.2 [31]. The maximum credibility clade tree and posterior probabilities were calculated using Tree Annotator v.1.10.4, applying a 25% burn-in. The Maximum Likelihood (ML) phylogenetic tree was obtained using IQ-TREE 2.2.2.6 [32]. The node support was calculated using both ultrafast bootstrap (UFBoot) [33] and the Shimodaira–Hasegawa-like approximate likelihood ratio test (SH-aLRT) [34], with 1000 replicates each.

### 2.5. Species Delimitation

We used a fragment of the mitochondrial gene cytochrome b (*CYTB*) to delimit species, instead of the more popular mitochondrial gene cytochrome *c* oxidase I (*COI*), which is often uninformative for inferring trichomycterine species limits, as discussed in [35]. Two coalescent single-locus, tree-based approaches for species delimitation were used: the Generalized Mixed Yule Coalescent (GMYC) [36] and the Bayesian implementation of the Poisson Tree Process (PTP) [37]. The GMYC was applied using both single and multiple thresholds. A dataset with one specimen per haplotype was used to meet the requirements of GMYC analyses, and the parameters to generate the ultrametric tree were presented in the previous section. The PTP was performed with 500,000 Markov chain Monte Carlo (MCMC) generations, thinning set to 100, a burn-in of 25%, *T. septemradiatus* set as the outgroup, and implementations of both Maximum Likelihood and Bayesian solutions. Both GMYC and PTP analyses were carried out on the Exelixis Lab’s web server (https://species.h-its.org/) (accessed on 25 May 2026). Additionally, a popular pairwise genetic distance-based method was also conducted, the Assemble Species by Automatic Partitioning (ASAP) [38], performed using the complete *CYTB* dataset alignment file and Jukes-Cantor (JC69) as a substitution model to compute the genetic distances. The calculations were obtained through the web server accessible at bioinfo.mnhn.fr/abi/public/asap/asapold.html. The optimal ASAP species delimitation was determined by the lowest ASAP partition score values. Results of molecular species delimitation were validated by concordant morphological data following [39]. This validation is necessary since genomic data alone can generate boundaries that may reflect genetic structure rather than species limits, a problem more likely to occur in single-locus analyses, thereby tending to inflate estimates of species diversity [39]. Therefore, we use morphological variation to validate genomic data, thus integrating distinct sources of evidence whose congruence corroborates hypotheses regarding species limits, a recommended practice for obtaining greater robustness in formulating hypotheses about species limits [39,40] and a central objective of the Integrative Taxonomy [41,42]. Such validation is particularly crucial in cases where different molecular approaches yield conflicting results concerning species limits. In this case, the molecular approach result that is congruent with morphological data will be preferred to delimit species. Delimitation of species with morphological data followed both the presence of unique derived character states found in a single species (e.g., autapomorphic criterion [40,43] and a unique combination of character states (i.e., the diagnosticability criterion [40,44]).

### 2.6. Delimitation of Areas of Endemism and Priority Areas for Conservation

The term areas of endemism here adopted follows the classical term used in biogeographical studies, commonly employed for the congruent distribution pattern of two or more taxa (e.g., [45]). Areas of endemism were based on congruent distribution patterns of *Trichomycterus* in RPSB, the fish group with the largest number of endemic species in mountainous areas. In these areas, the congruent distribution is determined by the sympatric occurrence of species, causing evident distribution overlap. In a few cases where the distribution of a species extends slightly beyond the distribution area of other endemic species (e.g., the distribution of *T. nigroauratus*), the area of endemism considered here is the largest area shared by at least two endemic species. The only species recorded for a wider region in the upper and middle RPSB, nearly overlapping the distribution of most other congeners, *T. paquequerense*, was considered a geographically widespread species not informative for delimiting areas of endemism.

The selection of priority areas for conservation was primarily determined by the number of endemic species and secondarily by the number of endangered species. The conservation status of *Trichomycterus* species represents the authors’ independent assessment following the categories and criteria proposed by the International Union for Conservation of Nature (IUCN) [46], rather than official IUCN Red List assessments. The extent of occurrence (EOO) was determined using the minimum convex polygon drawn around each location where each species is known to occur. The total area of each micro-basin was calculated using QGIS LTR 3.40 [47] with the HydroBASINS dataset [48]. The area of occupancy (AOO) was calculated using a 2 km x 2 km cell grid implemented in QGIS LTR 3.40.

## 3. Results

### 3.1. Species Delimitation

**Figure 1:**
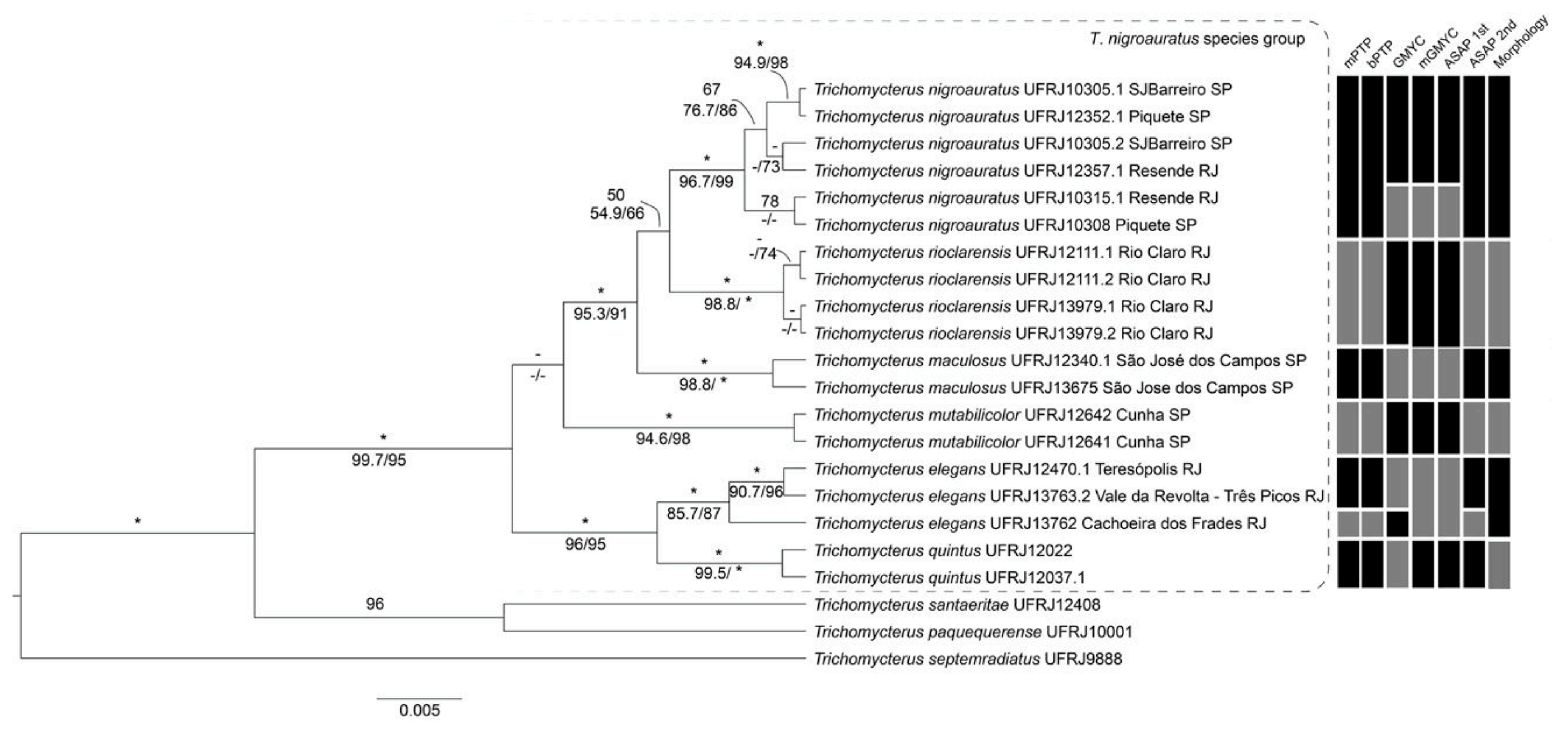
Ultrametric phylogenetic tree using sequences of the mitochondrially encoded cytochrome b gene (1078 bp) to delimit species of the *Trichomycterus nigroauratus* species group. Numbers above branches represent posterior probability values generated by Bayesian Inference, whereas numbers below branches represent Ultrafast Bootstrap and Shimodaira–Hasegawa-like approximate likelihood ratio test values. Taxon names are followed by UFRJ catalogue numbers and localities. ASAP 1st and 2nd, lowest ASAP partition score values; GMYC and mGMYC, Generalized Mixed Yule-Coalescent using single and multiple thresholds, respectively; mPTP and bPTP, Maximum Likelihood solution and Bayesian implementation of Poisson Tree Process, respectively. Asterisks (*) indicate maximum support values and dashes (-) indicate values below 50.

The molecular analyses of species delimitation provided results that were largely congruent with each other (Figure 1). All nominal species were supported as exclusive lineages with maximum values. Species limits for *T. maculosus*, *T. mutabilicolor* and *T. quintus* were unanimously corroborated in all analyses.

The limits previously proposed for *T. nigroauratus* [8] were not supported in three of the six molecular approaches, with both GMYC analyses and ASAP 1st indicating two different species (Figure 1). On the other hand, both PTP analyses and ASAP 2nd recognized a single species, which is concordant with morphological data. Therefore, following the proposed methodology using congruence between different sources of evidence as a main factor to delimit species, the result presented by GMYC analyses and ASAP 1st is rejected.

The analyses indicated the existence of undescribed species. The first one, *Trichomycterus rioclarensis* Costa, Barbosa, Katz, Vilardo, Mattos & Amorim sp. nov., endemic to the Rio Claro drainage, was supported in all molecular analyses (Figure 1) and corroborated by morphological features (see Taxonomic accounts below).

Within the lineage from the Rio Paquequer drainage, comprising populations from Cachoeira dos Frades, Teresópolis and Vale da Revolta, PTP analyses, GMYCs, and ASAP 2nd indicated two distinct undescribed species, one in Cachoeira dos Frades, and another in Teresópolis and Vale da Revolta (Figure 1). On the other hand, GMYCm and ASAP 1st indicated only one in the Rio Paquequer drainage (Figure 1). Morphological data corroborated a single species in the whole Rio Paquequer drainage, as supported by GMYCm and ASAP 2nd. Considering congruence of two molecular approaches, GMYCm and ASAP 2nd, with morphological data, we recognize a single species, *Trichomycterus elegans* Costa, Barbosa, Katz, Vilardo, Mattos & Amorim sp. nov.

### 3.2. Taxonomic Accounts

*Trichomycterus elegans* Costa, Barbosa, Katz, Vilardo, Mattos & Amorim sp. nov.

urn:lsid:zoobank.org:act: 770DF24A-B60C-4CD3-B106-5281664E5EEA 

**Figure 2:**
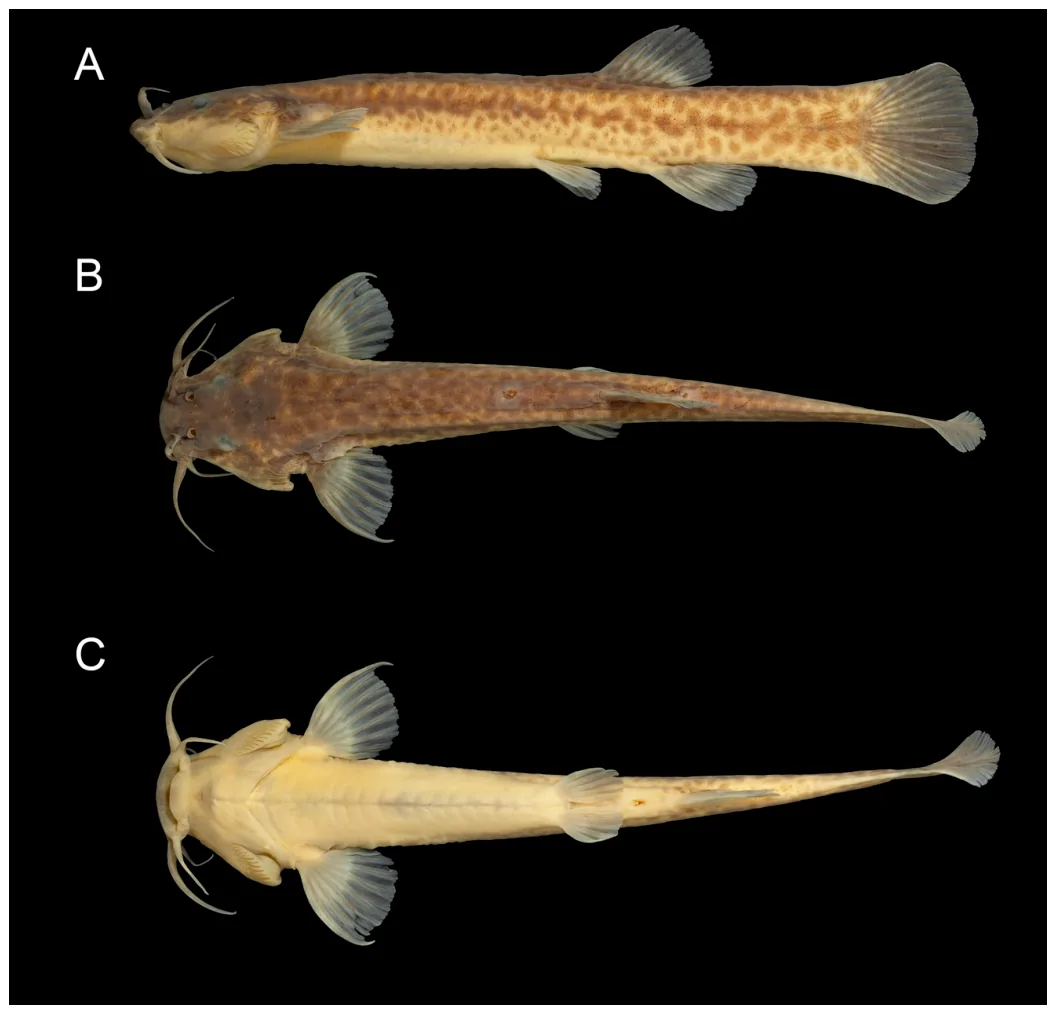
*Trichomycterus elegans* Costa, Barbosa, Katz, Vilardo, Mattos & Amorim sp. nov., holotype, UFRJ 14765, 56.0 mm SL. (**A**) lateral view; (**B**) dorsal view; (**C**) ventral view.

**Figure 3:**
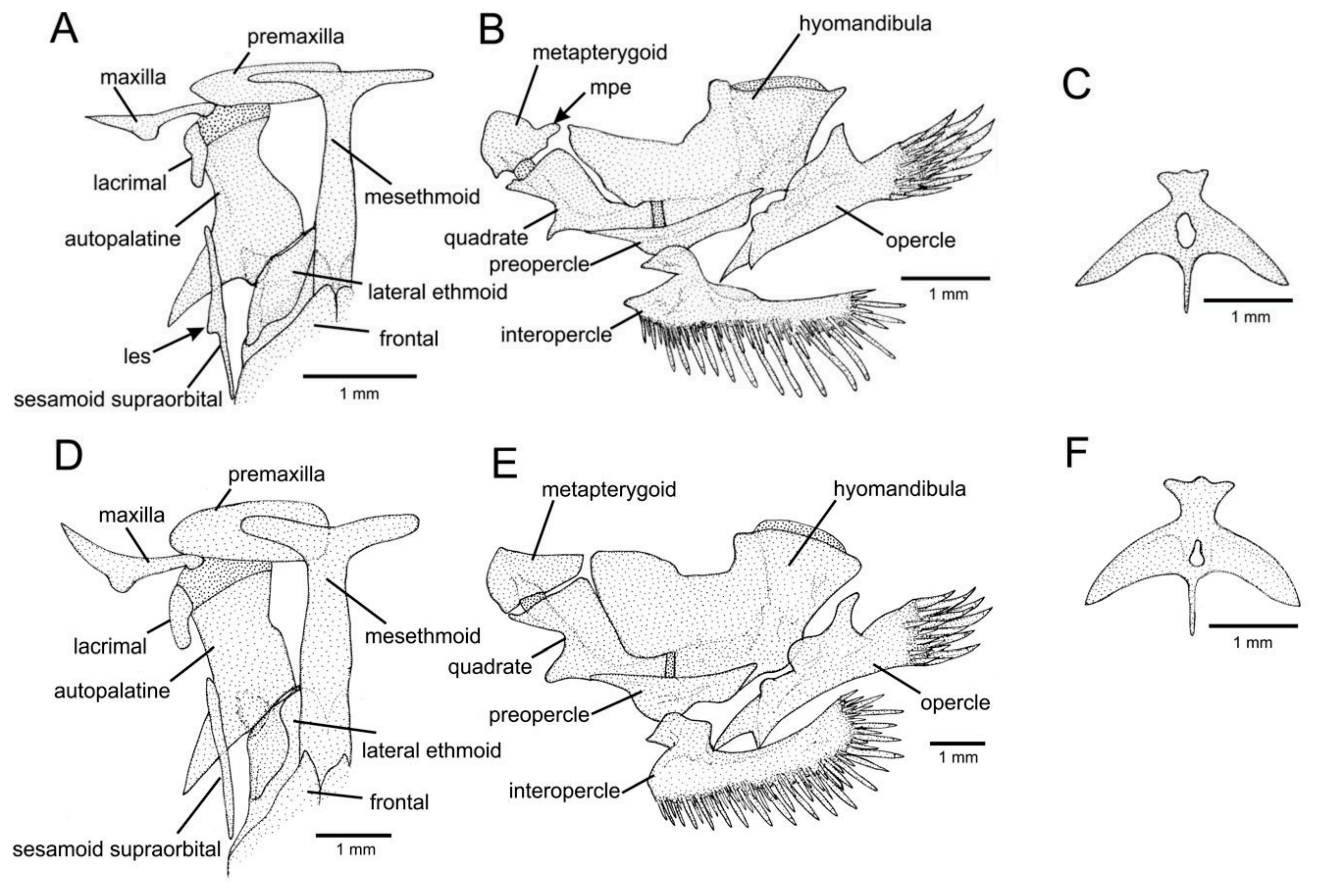
Osteological structures of: (**A**–**C**) *Trichomycterus elegans* Costa, Barbosa, Katz, Vilardo, Mattos & Amorim sp. nov.; (**D**–**F**) *Trichomycterus rioclarensis* Costa, Katz, Vilardo, Mattos & Amorim sp. nov.; (**A**,**D**) mesethmoidal region and adjacent structures, left and middle portions, dorsal view; (**B**,**E**) left jaw suspensorium and opercular series, lateral view; (**C**,**F**) parurohyal, ventral view. les, lateral expansion of sesamoid supraorbital; mpe, metapterygoid posterior expansion. Larger stippling represents cartilaginous areas.

Figure 2 and Figure 3A–C; Table 1

Holotype. UFRJ 14765, 56.0 mm SL; Brazil: Estado do Rio de Janeiro: Município de Teresópolis: Parque Nacional da Serra dos Órgãos (PARNASO) Rio Beija-Flor above dam in, Rio Paquequer drainage, which is part of the Rio Piabanha drainage, Rio Paraíba do Sul basin, 22°27′14″ S 42°59′34″ W, about 1070 m asl; S. Lima & C. Spada, 14 December 2002.

Paratypes. All from Brazil: Estado do Rio de Janeiro: Município de Teresópolis: Rio Paquequer drainage, which is part of the Rio Piabanha drainage, Rio Paraíba do Sul basin. UFRJ 14762, 9 ex., 16.7–37.8 mm SL; Poço Guarani, 22°26′48″ S 42°59′16″ W, about 985 m asl; S. Potsch & A. M Telles, 28 December 1983. UFRJ 4525, 3 ex., 40.8–41.2 mm SL; Rio Paquequer; M.C.C. de Pinna, 15 September 1985. UFRJ 1656, 1 ex., 49.6 mm SL; Farm Vale da Revolta; R. Sachsse, 16 November 1991. UFRJ 7041, 1 ex. (C&S), 38.4 mm SL; Vale da Revolta; S. Potsch et al., 16 June 1992. UFRJ 5626, 4 ex. (C&S), 27.2–36.8 mm SL; type locality; CICCAA 13620 (ex-UFRJ 5595), 3 ex., 48.6–51.9 mm SL; type locality; S. Lima & B. Filizola, 4 November 2002. UFRJ 5642, 2 ex., 45.3–51.4 mm SL; collected with holotype. UFRJ 12469, 6 ex., 33.1–40.7 mm SL; Poço do Castelo, PARNASO, 22°26′52″ S 42°59′03″ W, 930 m asl; A.M. Katz & P. Vilardo, 11 October 2019. UFRJ 14749, 3 ex. (C&S), 35.6–37.0 mm SL; type locality; A.M. Katz et al., 7 September 2025. UFRJ 13813, 2 ex., 46.5–45.7 mm SL; Cachoeira dos Frades, Rio dos Frades, affluent of Rio Preto, 22°20′21″ S 42°48′01″ W, 960 m asl; A.M. Katz et al., 30 June 2023. UFRJ 14757, 3 ex. (C&S), 41.0–45.1 mm SL; same locality as UFRJ 13813; A.M. Katz et al., 16 May 2025.

*Additional material (non-type specimens).* UFRJ 1142, 2 exs.; Teresópolis; M.C.C. de Pinna, 15 September 1985. UFRJ 1065, 1 ex.; Vale da Revolta; S. Potsch et al., 16 June 1992. UFRJ 7694, 1 ex.; type locality; I. de Farias, 31 August 2001. UFRJ 8123, 3 ex.; type locality; S. Lima & J. Valverde, 17 March 2002. UFRJ 5438, 23 ex.; UFRJ 5626, 4 ex. (C&S); type locality; S. Lima & B. Filizola, 27 April 2002. UFRJ 6112, 1 ex; Rio Canoas, 22°26′26″ S 42°53′02″ W, 890 m a.s.l.; F.P. Ottoni, 10 October 2004. UFRJ 6078, 2 ex.; Rio Canoas, 22°26′26″ S 42°53′02″ W, 890 m a.s.l.; F.P. Ottoni, 28 December 2004. UFRJ 7643, 8 ex.; Poço do Tatagiba, above the bridge of Rio Paquequer, PARNASO; H. Jabor et al., 29 May 2006. UFRJ 7643, 8 ex.; type locality, H. Jabor et al., 29 May 2006.

Diagnosis. *Trichomycterus elegans* is distinguished from all other species of TNG and subgenus *Trichomycterus* by having the medial margin of the autopalatine with a pronounced sinuosity (Figure 3A; vs. sinuosity weak or absent) and the presence of a distinctive narrow posterior extension in the metapterygoid (Figure 3B; vs. absence). *Trichomycterus elegans* also differs from all other species of TNG by the following combination of character states: 11–12 ventral procurrent caudal-fin rays (vs. 13–16 in *T. maculosus* and *T. mutabilicolor*); 37–38 free vertebrae (vs. 35–36 in *T. nigroauratus*, *T. quintus*, and *Trichomycterus rioclarensis* Costa, Katz, Vilardo, Mattos & Amorim sp. nov.); the base of the first dorsal-fin ray placed at the vertical through the centrum of the 19th or 20th vertebra (vs. 18th vertebra in *T. quintus* and *T. rioclarensis* Costa, Katz, Vilardo, Mattos & Amorim sp. nov.); 46–56 premaxillary teeth (vs. 65–68 in *T. quintus* and 70–74 in *T. rioclarensis* Costa, Katz, Vilardo, Mattos & Amorim sp. nov.); 43–60 dentary teeth (vs. 70–78 in *T. nigroauratus*, 76–86 in *T. rioclarensis* Costa, Katz, Vilardo, Mattos & Amorim sp. nov.); nasal barbel reaching area between orbit and opercular patch of odontodes (vs. reaching orbit or area anterior to it in *T. maculosus*); juvenile specimens with a black stripe along the longitudinal flank midline and adults with spotted flank (vs. all stages with the whole flank black in *T. quintus*); adult specimens about 50–60 mm SL with small round dark brown spots on the flank, larger spots about equal to opercular odontode patch in size (vs. with spots larger than interopercular odontode patch in *T. maculosus* and *T. nigroauratus*); presence of a ventro-lateral expansion on the middle portion of the sesamoid supraorbital, sometimes not visible in a dorsal view (Figure 3A; vs. absence in all other TNG species, Figure 3D, except *T. quintus*); and a deep concavity between the dorsal process and the main axis of the interopercle (Figure 3B; vs. a relatively shallow concavity in all other TNG species, Figure 3E, except *T. quintus*). *Trichomycterus elegans* is the smallest species of TNG, with the largest specimen measuring 56.0 mm SL (vs. about 70 mm SL or more).

Description. External morphology: Morphometric data in Table 1. Body slender, slightly compressed. Greatest body depth in area immediately anterior to pelvic-fin base. Dorsal profile of trunk slightly convex between nape and dorsal-fin origin, nearly straight on caudal peduncle. Ventral profile of trunk approximately straight. Head sub-trapezoidal in dorsal view, anterior profile of snout slightly convex. Axillary organ prominent, immediately below lateral line pores.

Eye large, oval, horizontal length longer than vertical length, dorsally positioned on head, about equidistant between snout tip and posterior margin of opercle. Distance between orbital rim and anterior nostril about half distance between anterior and posterior nostrils. Anterior nostril opening anterolaterally directed, surrounded by short membrane forming short tube, laterally continuous to nasal barbel. Posterior nostril opening dorsally directed, surrounded by short membrane, except on its posterior margin. Barbels moderate in length, tip of nasal barbel reaching area posterior to orbit, tip of maxillary barbel reaching area between middle and posterior portions of interopercular patch of odontodes and pectoral-fin base, rictal barbel reaching middle portion of interopercular patch of odontodes, or area immediately anterior to it. Mouth subterminal. Lateral fleshy lobes of mouth moderate, its largest length about one third or half lower jaw length excluding lobes. Jaw teeth pointed, arranged in three irregular rows, total of 46–56 premaxillary and 43–60 dentary teeth. Snout, lips and ventral surface of head covered by minute skin papillae. Branchial membrane thickened, attached to isthmus only at its anterior-most point, in ventral midline. Seven or eight branchiostegal rays. Opercular membrane not extending beyond limits of opercular patch of odontodes. Posterior limit of opercle patch of odontodes at vertical through pectoral-fin base. Opercular odontodes straight to slightly curved, arranged in oblique rows; interopercular odontodes narrow and pointed, arranged in three irregular rows. Seventeen to 20 opercular and 48–56 interopercular odontodes.

Lateral line canal of trunk short, restricted to anterior-most part of flank, with two pores, posteriorly followed by line of minute neuromasts until caudal-fin base. First lateral line pore immediately posterior to opercle. Supraorbital canal continuous, with three paired pores, s1, adjacent to medial margin of anterior nostril, s3, adjacent and just posterior to medial margin of posterior nostril, and s6, at transverse line through posterior portion of orbit. Pore s6 nearer its symmetrical homologous pore than orbit. Infraorbital canal represented by two short canal segments. Anterior infraorbital segment isolated from other cephalic canals, with two paired pores, i1, at transverse line through anterior nostril, and i3, at transverse line just anterior to posterior nostril. Series of seven or eight conspicuous neuromasts between pores i1 and s1, marginating anterior nostril. Posterior infraorbital segment with two pores, i10, adjacent to ventral margin of orbit, and i11, posterior to orbit. Series of about eight to 12 min infraorbital neuromasts anterior to posterior infraorbital segment. Postorbital canal with two pores, po1, at vertical through posterior portion of interopercular patch of odontodes, and po2, at vertical through opercular patch of odontodes. Supraorbital canal, posterior segment of infraorbital canal and postorbital canal interconnected.

Dorsal fin subtriangular, dorsal margin slightly rounded, dorsal-fin origin nearer caudal-fin posterior margin than snout. Twelve (iii + III + 7) dorsal-fin rays. Anal fin smaller than the dorsal fin, subtriangular, ventral margin slightly rounded. Anal-fin origin at vertical through base of last two dorsal-fin rays or posterior to it. Ten or 11 (iii–iv + II + 5) anal-fin rays. Pectoral fin subtriangular in dorsal view, first ray terminating in short filament about 10% of pectoral-fin length. Eight (I + 7) pectoral-fin rays. Pelvic fin sub-oval, its posterior extremity reaching vertical through area just anterior to middle of dorsal-fin base. Pelvic fin tip not reaching anus; anus and urogenital papilla opening at vertical through area just anterior to middle of dorsal-fin base. Pelvic-fin bases medially separated by narrow interspace. Five (I + 4) pelvic-fin rays. Caudal fin subtruncate with posterior corners rounded, posterior margin slightly convex. Thirteen (I + 11 + I) principal caudal-fin rays, 15–16 (xiv–xv + I) dorsal procurrent rays, and 11–13 (x–xii + I) ventral procurrent rays.

Osteology (Figure 3A–C): Mesethmoid thin, T-shaped, cornu narrow, with rounded extremity. Premaxilla relatively long and narrow, sub-rectangular, its largest length about equal autopalatine length, excluding postero-lateral process. Maxilla long, slightly curved, almost straight, shorter than premaxilla, with small posterior process. Lacrimal flat, sub-elliptical, supporting anterior infraorbital canal. Sesamoid supraorbital, rod-shaped, narrower than sesamoid supraorbital, with lateral triangular expansion, about 2.5 times longer than lacrimal. Lacrimal separated from sesamoid supraorbital by interspace shorter than lacrimal longitudinal length.

Autopalatine sub-rectangular in dorsal view when excluding postero-lateral process, lateral margin slightly concave, medial margin sinuous, with deep anterior concavity posteriorly followed by convexity. Autopalatine postero-lateral process moderate in length subtriangular, its longest length about twothirds autopalatine length, excluding postero-lateral process. Metapterygoid relatively small subtriangular, anterior margin convex posterior margin with distinctive narrow posterior extension. Quadrate robust, posterodorsal margin in close proximity to anterior hyomandibula outgrowth. Hyomandibula long, occupying about three-fourths of jaw suspensorium length.

Opercle slender, nearly so long as interopercle. Opercular odontode patch moderate, its depth about three-fifths of hyomandibula articular facet length. Dorsal process of opercle small, with distinctive dorsal facet for insertion of *dilatator operculi* muscle. Opercular articular facet for hyomandibula with rounded laminar projection. Articular facet for preopercle small. Interopercle long, its largest length slightly shorter than hyomandibula horizontal length. Preopercle small, with weak ventral projection.

Parurohyal robust anteriorly, with small paired anterolateral process, thin posteriorly. Lateral process relatively short, slightly curved, pointed. Posterior process narrow and moderate in length, about half lateral process length.

Caudal skeleton comprising two dorsal hypural plates, corresponding to hypurals 4 + 5 and 3, respectively, and single ventral hypural plate corresponding to hypurals 1 and 2 and parhypural. Base of first dorsal-fin ray at vertical through centrum of 19th or 20th vertebra, base of first anal-fin ray at vertical through centrum of 23rd or 24th vertebra. Thirty-seven or 38 vertebrae and 13 ribs.

Colouration in alcohol (Figure 2): Flank and dorsum pale brownish yellow, with small rounded brown spots, darker on longitudinal midline of flank; larger spots slightly larger than orbit. Venter yellowish white. Dorsal and dorsolateral regions of head dark brown, ventral and ventrolateral regions yellowish white. Nasal and maxillary barbels dark brown, rictal barbel yellowish white. Fins hyaline with pale brown rays; brown spots on basal portion of unpaired fins. In specimens below about 35 mm SL, flank with broad black stripe on longitudinal midline.

Etymology. From the Latin *elegans*, meaning fine, elegant, in reference to the slender body morphology of the new species.

**Figure 4:**
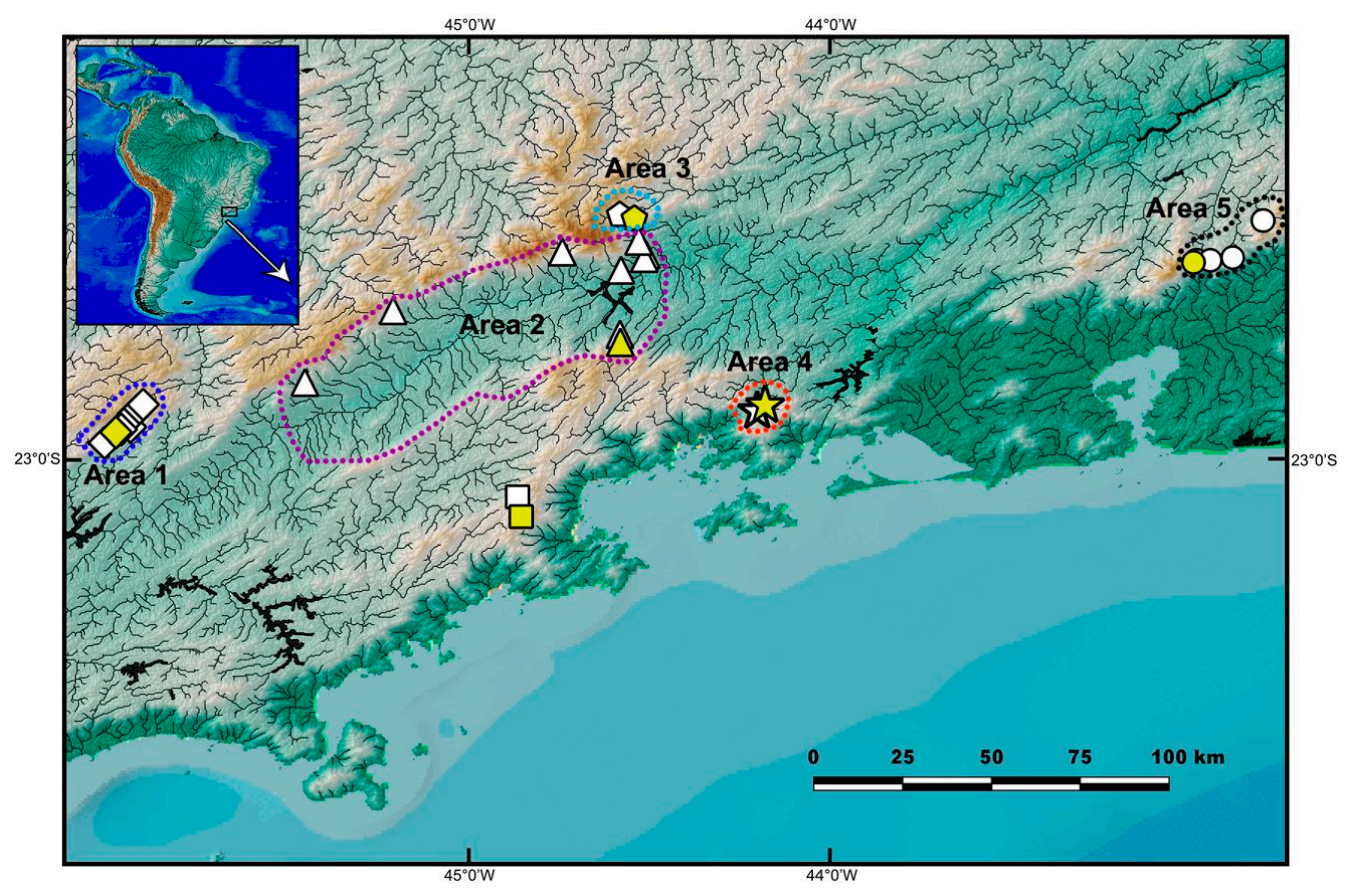
Geographical distribution of the *Trichomycterus nigroauratus* group. Lozenge, *Trichomycterus maculosus*; triangles, *Trichomycterus nigroauratus*; squares, *Trichomycterus mutabilicolor*; stars, *Trichomycterus rioclarensis* Costa, Katz, Vilardo, Mattos & Amorim sp. nov.; circles, *Trichomycterus elegans* Costa, Barbosa, Katz, Vilardo, Mattos & Amorim sp. nov. Yellow symbols are type localities.

Distribution. *Trichomycterus elegans* occurs in the upper Rio Paquequer and upper Rio dos Frades drainages, Rio Paraiba do Sul basin, southeastern Brazil, in altitudes between about 930 and 1070 m asl (Figure 4).

*Trichomycterus rioclarensis* Costa, Katz, Vilardo, Mattos & Amorim sp. nov.

urn:lsid:zoobank.org:act: 66045C67-CD87-4302-85F7-2FBBE3E6DA2B 

**Figure 5:**
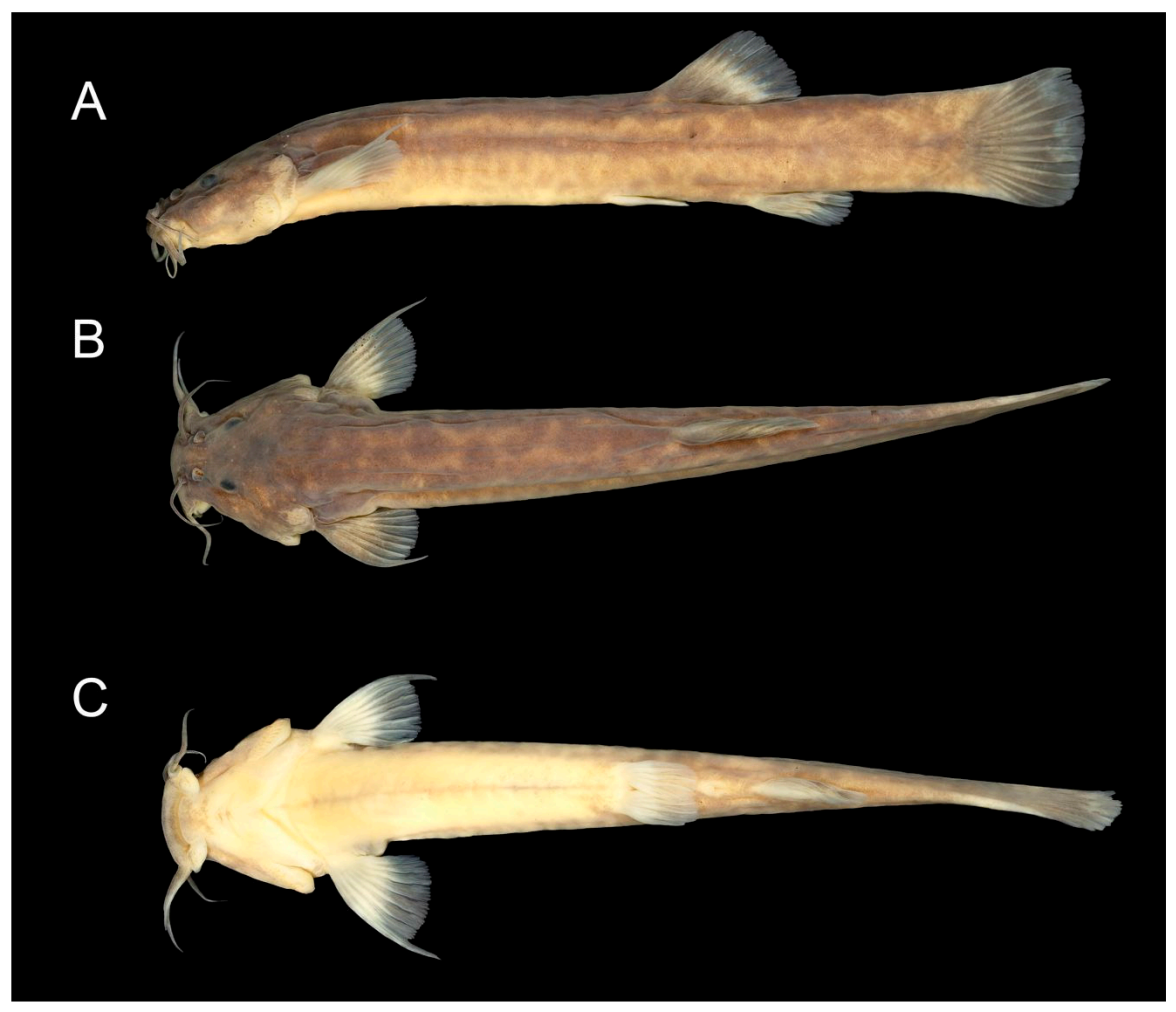
*Trichomycterus rioclarensis* Costa, Katz, Vilardo, Mattos & Amorim sp. nov., holotype, UFRJ 14766, 62.9 mm SL. (**A**) lateral view; (**B**) dorsal view; (**C**) ventral view.

Figure 3D–F and Figure 5; Table 2

*Trichomycterus nigroauratus* non *Trichomycterus nigroauratus Barbosa & Costa*, 2008: [49]: 43 (mididentification).

Holotype. UFRJ 14766, 62.9 mm SL; Brazil: Estado do Rio de Janeiro: Município de Rio Claro: Rio Parado above the village of Lídice, Rio Piraí drainage, Rio Paraíba do Sul basin, 22°50′59″ S 44°10′47″ W, about 550 m asl; J.L. Mattos et al., 14 July 2023.

Paratypes. All from Brazil: Estado do Rio de Janeiro: Município de Rio Claro: Rio Piraí drainage, Rio Paraíba do Sul basin. UFRJ 12105, 3 ex. (C&S), 40.9–71.5 mm SL; type locality; B. Mesquita et al., 21 December 2018. UFRJ 12106, 1 ex, 45.8 mm SL; type locality; B. Mesquita et al., 22 December 2018. UFRJ 13953, 1 ex., 31.0 mm SL; UFRJ 13959, 7 ex., 28.2–38.9 mm SL; CICCAA 13621; 3 ex., 29.4–35.2 mm SL; collected with holotype. UFRJ 12094, 1 ex: Rio das Pedras, 22°51′59″ S 44°12′07″ W, about 555 m asl; B. Mesquita et al., 21 December 2018.

Diagnosis. *Trichomycterus rioclarensis* is distinguished from all other congeners of TNG by having: a relatively broad premaxilla (Figure 3D), with 70–74 teeth arranged in six irregular rows (vs. premaxilla moderately narrow, Figure 3A, with 46–68 teeth arranged in three or four irregular series); and dentary teeth arranged in four irregular rows (vs. three).

*Trichomycterus rioclarensis* also differs from other congeners of TNG by the following combination of character states: 10–12 procurrent caudal-fin rays (vs. 13–14 in *T. quintus* and 13–16 in *T. mutabilicolor*); 36 vertebrae (vs. 37–39 in *T. elegans* and *T. mutabilicolor*); 55–60 interopercular odontodes (vs. 45–50 in *T. mutabilicolor*); base of the first dorsal-fin ray at a vertical through the centrum of the 18th vertebra (vs. between the 19th and 20th in *T. elegans* and between the 20th and 21st in *T. mutabilicolor*); 76–86 dentary teeth (vs. 43–68 in *T. elegans*, *T. maculosus*, *T. mutabilicolor* and *T. quintus*); the nasal barbel reaching area posterior to the orbit (vs. reaching orbit or area anterior to it in *T. maculosus*); juvenile specimens with a black stripe along the longitudinal flank midline and adults with spotted flank (vs. all stages with the whole flank black in *T. quintus*); larger adult specimens above about 65 mm SL with flank predominantly dark grey to black, with almost inconspicuous dark marks (Figure 5; vs. larger specimens with conspicuous dark spots over lighter background in *T. maculosus*, *T. mutabilicolor* and *T. nigroauratus*); the absence of a ventro-lateral expansion on the middle portion of the sesamoid supraorbital, sometimes not visible in a dorsal view (Figure 3D; vs. the presence in *T. elegans* and *T. quintus*, Figure 3A); and a relatively shallow concavity between the dorsal process and the main axis of the interopercle (Figure 3E; vs. a deep concavity in *T. elegans* and *T. quintus*, Figure 3B).

Description. External morphology: Morphometric data in Table 2. Body moderately slender, slightly compressed. Greatest body depth in area immediately anterior to pelvic-fin base. Dorsal profile of trunk slightly convex between nape and dorsal-fin origin, nearly straight on caudal peduncle. Ventral profile of trunk approximately straight. Head sub-trapezoidal in dorsal view, anterior profile of snout slightly convex. Axillary organ prominent, immediately below lateral line pores.

Eye large, oval, horizontal length longer than vertical length, dorsally positioned on head, about equidistant between snout tip and posterior margin of opercle. Distance between orbital rim and anterior nostril about one and half times distance between anterior and posterior nostrils. Anterior nostril opening anterolaterally directed, surrounded by short membrane forming short tube, laterally continuous to nasal barbel. Posterior nostril opening dorsally directed, surrounded by short membrane, except on its posterior margin. Barbels moderate in length, tip of nasal barbel reaching area posterior to orbit, tip ofmaxillary barbel reaching area between middle and posterior portions of interopercular patch of odontodes, rictal barbel reaching anterior portion of interopercular patch of odontodes, or area immediately anterior to it.

Mouth subterminal. Lateral fleshy lobes of mouth large, its largest length about two-thirds of lower jaw excluding lobes. Premaxillary teeth arranged in six irregular rows, dentary teeth arranged in four irregular rows. Teeth of external row incisiform, teeth of middle rows sub-incisiform with slightly rounded tips, teeth of internal-most rows sharply pointed. Total of 70–74 premaxillary and 76–86 dentary teeth.

Snout, lips and ventral surface of head covered by minute skin papillae. Branchial membrane thickened, attached to isthmus only at its anterior-most point, in ventral midline. Seven branchiostegal rays. Opercular membrane not extending beyond limits of opercular patch of odontodes. Posterior limit of opercular patch of odontodes at vertical through pectoral-fin base. Opercular odontodes conical, straight to slightly curved, irregularly arranged in specimens above 50 mm SL, arranged in oblique rows in smaller specimens. Interopercular odontodes arranged in three irregular rows, narrow and pointed in external rows, longer, broader and with rounded tips in internal-most row. Total of 18–20 opercular and 55–60 interopercular odontodes.

Lateral line canal of trunk short, restricted to anterior-most part of flank, with two pores, posteriorly followed by line of minute neuromasts until caudal-fin base. First lateral line pore immediately posterior to opercle. Supraorbital canal continuous, with three paired pores, s1, adjacent to medial margin of anterior nostril, s3, adjacent and just posterior to medial margin of posterior nostril, and s6, at transverse line through posterior portion of orbit. Pore s6 nearer its symmetrical homologous pore than orbit. Infraorbital canal represented by two short canal segments. Anterior infraorbital segment isolated from other cephalic canals, with two paired pores, i1, at transverse line through anterior nostril, and i3, at transverse line just anterior to posterior nostril. Series of eight or nine conspicuous neuromasts between pores i1 and s1, marginating anterior nostril. Posterior infraorbital segment with two pores, i10, adjacent to ventral margin of orbit, and i11, posterior to orbit. Series with about 20 min infraorbital neuromasts, anterior to posterior infraorbital canal segment. Postorbital canal with two pores, po1, at vertical through posterior portion of interopercular patch of odontodes, and po2, at vertical through opercular patch of odontodes. Supraorbital canal, posterior segment of infraorbital canal and postorbital canal interconnected.

Dorsal fin subtriangular, dorsal margin nearly straight, dorsal-fin origin nearer caudal-fin posterior margin than snout. Twelve (iii + III + 7) dorsal-fin rays. Anal fin smaller than the dorsal fin, subtriangular, ventral margin about straight. Anal-fin origin at vertical through base of last dorsal-fin ray or posterior to it. Ten (iii + II + 5) anal-fin rays. Pectoral fin subtriangular in dorsal view, first ray terminating in filament about 30–40% of pectoral-fin length. Eight (I + 7) pectoral-fin rays. Pelvic fin sub-oval, its posterior extremity reaching vertical through middle of dorsal-fin base. Pelvic fin tip not reaching anus; anus and urogenital papilla opening at vertical through area just posterior to middle of dorsal-fin base. Pelvic-fin bases medially separated by narrow interspace. Five (I + 4) pelvic-fin rays. Caudal fin subtruncate with posterior corners rounded, posterior margin approximately straight. Thirteen (I + 11 + I) principal caudal-fin rays, 13–15 (xii–xiv + I) dorsal procurrent rays, and 10–12 (ix–xi + I) ventral procurrent rays.

Osteology (Figure 3D–F): Mesethmoid moderately thin to thick in largest specimen, T-shaped, cornu moderately narrow to broad, with rounded extremity. Premaxilla relatively long and broad, sub-rectangular, its largest length about equal autopalatine length excluding postero-lateral process. Maxilla long, weakly curved, slightly shorter than premaxilla, with small posterior process. Lacrimal flat, sub-elliptical, supporting anterior infraorbital canal. Sesamoid supraorbital rod-shaped, without lateral expansions, narrower than sesamoid supraorbital, about 2.5 times longer than lacrimal. Lacrimal separated from sesamoid supraorbital by interspace shorter than lacrimal longitudinal length.

Autopalatine sub-rectangular in dorsal view when excluding postero-lateral process, lateral margin about straight, medial margin straight to weakly sinuous. Autopalatine postero-lateral process moderate in length, subtriangular, its longest length about two-thirds autopalatine length excluding postero-lateral process. Metapterygoid moderate in size, subtriangular, posteriorly elongated, anterior margin convex, postero-dorsal margin slightly concave, almost straight. Quadrate robust, posterodorsal margin in close proximity to anterior hyomandibula outgrowth. Hyomandibula long, occupying about three-fourths of jaw suspensorium length.

Opercle slender, slightly longer than interopercle. Opercular odontode patch moderate, its depth about three-quarters of hyomandibula articular facet length. Dorsal process of opercle small, with distinctive dorsal facet for insertion of *dilatator operculi* muscle. Opercular articular facet for hyomandibula with prominent rounded laminar projection. Articular facet for preopercle small. Interopercle long, its largest length about four-fifths of hyomandibula outgrowth horizontal length. Preopercle small, with small ventral projection.

Parurohyal robust anteriorly, with small paired anterolateral process, thin posteriorly. Lateral process relatively short, slightly curved, pointed. Posterior process narrow and moderate in length, about half lateral process length.

Caudal skeleton comprising two dorsal hypural plates, corresponding to hypurals 4 + 5 and 3, respectively, and single ventral hypural plate, corresponding to hypurals 1 and 2 and parhypural. Base of first dorsal-fin ray at vertical through centrum of 18th vertebra, base of first anal-fin ray at vertical through centrum of 23rd vertebra. Thirty-six vertebrae and 13 ribs.

Colouration in alcohol (Figure 5): Flank, dorsum and head side brown with diffuse darker areas. Nasal and maxillary barbels brown, rictal barbel grey. Venter yellowish white. Unpaired fins dark brown. Paired fins hyaline, basal portion of pectoral fin pale brown. In specimens below about 35 mm SL, flank with broad black stripe on longitudinal midline.

Etymology. The name *rioclarensis* is an allusion to the occurrence of the new species in the Município of Rio Claro, southeastern Brazil.

Distribution. Upper Rio Piraí drainage, middle Rio Paraíba do Sul basin, southeastern Brazil (Figure 4).

### 3.3. Areas of Endemism

Each of the six species of the TNG here delimited occurs in a single disjunct mountainous area of the upper and middle sections of RPSB (Figure 4). No species of TNG was found during our intensive field inventories in intermediate areas, nor in tributaries of the lower section of the RPSB such as Rio Grande and Rio Pomba drainages. Five of these six areas are greatly overlapped and nearly coincident with the distribution areas of congeners of other species groups, comprising the *Trichomycterus itatiayae* species group of the subgenus *Paracambeva* Costa, 2021 [5], the *Trichomycterus mirissumba* group of the subgenus *Cryptocambeva* Costa, 2021 [13], the *Trichomycterus goeldii* and *Trichomycterus travassosi* groups of the subgenus *Psammocambeva* Costa, 2021 [4], and the subgenus *Humboldtglanis* Costa, 2021 [3], thus supporting five areas of endemism (Table 3).

## 4. Discussion

Areas of endemism, besides being basic units in biogeographical studies [50], play a pivotal role in the selection of priority areas for biodiversity conservation [51,52]. The recognition of areas of endemism gains special importance in regions with high biodiversity and marked environmental decline, as is the case of the Atlantic Forest of eastern South America, one of the main biodiversity hotspots in the world [1]. Despite the presence of protected areas in RPSB (Table 3), our field studies have shown that the region is still highly impacted by agriculture, deforestation, livestock, domestic sewage and the introduction of trout. Therefore, identifying riverine areas of endemism that represent biodiversity centres is important for focusing efforts to protect the riverine fauna.

The two species of TNG occurring in areas with greater concentration of endemic trichomycterines are *T. nigroauratus* and *T. quintus*, the former species occurring in a broad region of the upper-middle RPSB in the Serra da Mantiqueira and Serra da Bocaina, overlapping the distributional limits of the other four congeners (Area 2 in Table 3), and the latter species endemic to the upper Rio Preto drainage in the Serra da Mantiqueira in an area also inhabited by the other three endemic congeners (Area 3 in Table 3). Despite the geographical proximity between Areas 2 and 3 (Figure 4), they are not directly connected by river drainages. These two areas are separated by a branch of the Serra da Mantiqueira (i.e., the Serra da Pedra Selada), with elevations between about 1100 and 1600 m asl. The Rio Preto drainage of Area 3 is a left-bank tributary of the lower-middle RPSB, whereas rivers draining Area 2 are connected to the upper-middle section of the basin.

The great concentration of endemic species in Areas 2 and 3, with five and four endemic species, respectively, supports them as priority areas for conservation. These areas together correspond to 56% of the total number of endemic species occurring in the five areas (Table 3). Area 3 also deserves special attention by sheltering two species here considered endangered. Since Areas 2 and 3 are geographically contiguous areas in the Serra da Mantiqueira (Figure 4), they should be considered as a single biodiversity hotspot for trichomycterine catfishes in the Atlantic Forest of southeastern Brazil and therefore a priority area for conservation of the aquatic biodiversity.

## 5. Conclusions

Financial resources for species conservation are generally limited, especially in developing countries. For this reason, strategies for identifying conservation priorities are fundamental to establishing policies that effectively protect biodiversity. The approach followed in this study, combining Integrative Taxonomy to delimit areas of endemism with criteria for assessing conservation status, holds great potential as a method for selecting priority areas for biodiversity conservation.

## Figures and Tables

**Table 1:** Morphometric data of *Trichomycterus elegans* Costa, Barbosa, Katz, Vilardo, Mattos & Amorim sp. nov.

	Holotype	Paratypes (n = 7)		
				
Standard length (SL)	56.0	33.3–51.4	Mean	SD
				
Percentage of standard length				
Body depth	12.5	12.8–15.3	13.7	1.03
Caudal peduncle depth	10.7	10.6–12.5	11.2	0.62
Body width	8.8	9.8–11.3	10.2	0.71
Caudal peduncle width	2.5	2.5–4.0	3.1	0.49
Pre-dorsal length	62.5	61.3–66.4	62.8	1.6
Pre-pelvic length	55.5	55.3–63.4	57.4	2.81
Dorsal-fin base length	10.6	11.0–12.3	11.6	0.65
Anal-fin base length	9.4	8.3–9.9	9.0	0.51
Caudal-fin length	15.1	15.7–17.8	16.5	0.95
Pectoral-fin length	14.5	12.5–15.7	14.4	0.91
Pelvic-fin length	10.2	9.7–11.3	10.3	0.54
Head length	21.0	22.1–24.5	22.7	1.09
				
Percentage of head length				
Head depth	47.2	43.3–49.5	45.4	2.14
Head width	91.4	79.8–91.1	86.4	4.54
Snout length	46.0	23.9–47.7	42.1	7.53
Interorbital width	31.1	27.6–42.8	31.1	4.95
Preorbital length	14.5	12.1–23.8	14.8	3.8
Eye diameter	14.7	14.1–18.1	15.2	1.24

**Table 2:** Morphometric data of *Trichomycterus rioclarensis* Costa, Katz, Vilardo, Mattos & Amorim sp. nov.

	Holotype	Paratypes (n = 7)		
				
Standard length (SL)	62.9	31.0–65.0	Mean	SD
				
Percentage of standard length				
Body depth	12.5	13.2–16.4	14.4	1.24
Caudal peduncle depth	11.6	10.9–12.2	11.6	0.49
Body width	10.6	10.4–12.1	11.2	0.72
Caudal peduncle width	3.2	2.8–3.9	3.0	1.25
Pre-dorsal length	58.8	61.4–63.6	62.1	1.48
Pre-pelvic length	56.6	56.7–59.3	57.9	0.95
Dorsal-fin base length	12.1	11.3–13.4	12.1	0.64
Anal-fin base length	8.8	8.9–9.8	9.2	0.4
Caudal-fin length	15.2	16.0–17.6	16.6	0.73
Pectoral-fin length	12.6	13.7–15.2	14.3	0.83
Pelvic-fin length	8.5	9.4–11.9	10.6	1.19
Head length	20.2	21.3–24.1	22.4	1.27
				
Percentage of head length				
Head depth	48.1	42.9–46.3	45.0	1.7
Head width	87.8	77.4–86.0	83.5	3.23
Snout length	42.9	40.0–44.9	42.8	1.8
Interorbital width	29.3	24.0–30.6	26.6	2.44
Preorbital length	13.7	10.2–13.8	12.3	1.4
Eye diameter	12.7	13.1–16.4	14.9	1.62

**Table 3:** Areas of endemism of endemic trichomycterine and authors’ conservation status assessment using categories and criteria described in IUCN guidelines. AOO, area of occupancy; CA, category (EN, endangered, LC, low concern; NT, near threatened); CR, criteria used to classify threatened species; EOO, extent of occurrence; NL, number of locations; OPA, occurrence in protected areas (BO, Parque Nacional da Bocaina; IT, Parque Nacional de Itatiaia; PP, Parque Estadual da Pedra Selada; TP, Parque Estadual Três Picos; SO, Parque Nacional da Serra dos Órgãos); REF, main references (PS, present study). Species names in bold refer to species of the *T. nigroautaus* group.

	Endemic Species of *Trichomycterus*	REF	OPA	NL	AOO	EOO	CA	CR
1 (upper Rio do Peixe drainage)	***T. (Trichomycterus) maculosus* Barbosa & Costa, 2010**	PS	No	8	32 km^2^	321.06 km^2^	LC	
	*T. (Paracambeva) antiquus* Costa, Feltrin, Mattos & Katz, 2024	[7]	No	2	8 km^2^	165.81 km^2^	NT	B1a; B2a
								
2 (upper-middle Rio Paraíba do Sul basin)	***T. (Trichomycterus) nigroauratus* Barbosa & Costa, 2008**	PS	BO, IT	12	28 km^2^	6050.6 km^2^	LC	
	*T. (Cryptocambeva) mariamole* Barbosa & Costa, 2010	[15]	BO, IT	4	8 km^2^	5523.75 km^2^	NT	B1abiii; B2abiii
	*T. (Cryptocambeva) mimonha* Costa, 1992	[14]	No	2	4 km^2^	212.98 km^2^	NT	B1abiii; B2abiii
	*T. (Paracambeva) itatiayae* Miranda-Ribeiro, 1906	[7]	BO, IT	5	16 km^2^	817.11 km^2^	LC	
	*T. (Psammocambeva) travassosi* (Miranda Ribeiro, 1949)	[6]	BO, IT	4	16 km^2^	575.45 km^2^	LC	
								
3 (upper Rio Preto drainage)	***T. (Trichomycterus) quintus* Costa, 2020**	PS	No	2	8 km^2^	135.47 km^2^	LC	
	*T. (Cryptocambeva) mirissumba* Costa, 1992	[14]	IT, PP	6	16 km^2^	270.99 km^2^	EN	B1abiii; B2abiii
	*T. (Humboldtglanis) albinotatus* Costa, 1992	[8]	IT, PP	11	28 km^2^	512.66 km^2^	EN	B2abiii
	*T. (Psammocambeva) auroguttatus* Costa, 1992	[6]	No	7	20 km^2^	135.47 km^2^	LC	
								
4 (upper Rio Piraí drainage)	***T. (Trichomycterus) rioclarensis* Costa, Katz, Vilardo, Mattos & Amorim sp. nov.**	PS	No	5	16 km^2^	243.32 km^2^	LC	
	*T. (Cryptocambeva) claudiae* Barbosa & Costa, 2010	[15]	No	4	16 km^2^	703.33. km^2^	NT	B1a; B2a
	*T. (Psammocambeva) macrophthalmus* Barbosa & Costa, 2010	[6]	No	4	16 km^2^	1065.46 km^2^	LC	
								
5 (upper Rio Paquequer drainage)	***T. (Trichomycterus) elegans* Costa, Barbosa, Katz, Vilardo, Mattos & Amorim sp. nov.**	PS	SO, TP	7	24 km^2^	824.18 km^2^	LC	
	*T. (Psammocambeva) goeldii* Boulenger, 1896	[6]	SO, TP	11	36 km^2^	1497.65 km^2^	LC	

## Data Availability

DNA sequences used in this study were deposited in GenBank (see Appendix A for access codes).

## Appendix A

### Comparative Material – H, Holotype; P, Paratypes

***Trichomycterus maculosus* Barbosa & Costa, 2010**

All from Brazil: Estado de São Paulo: Município de São José dos Campos: Rio do Peixe drainage, Rio Paraíba do Sul basin. UFRJ 6033, holotype; UFRJ 5693, 2 paratypes; Cachoeira Pedro David, near village of São Francisco Xavier, 22°55′30″ S 45°58′50″ W, about 800 m asl; W.J.E.M. Costa, B.B. Costa & C.P. Bove, 23 January 2003. UFRJ 677, 6 paratypes; UFRJ 5168, 1 paratype (C&S); UFRJ 5169, 2 paratypes (C&S); same locality as holotype; W.J.E.M. Costa & C.P. Bove, 17 March 1991. UFRJ 13023, 1 ex.; São Francisco Xavier, 22°57′14″ S 46°00′44″ W, about 860 m asl W; A.M. Katz et al., 15 August 2019. UFRJ 13367, 3 ex. (DNA); UFRJ 13368, 5 ex.; confluence of Riacho Santa Bárbara with Rio do Peixe, 22°54′37″ S 45°56′24″ W, about 715 m asl; C.R.M. Feltrin; 16 December 2021. UFRJ 13675, 1 ex. (DNA); upper section of Rio Santa Bárbara, tributary of Rio do Peixe: 22°52′34″ S 45°56′02″ W; about 1275 m asl; C.R.M. Feltrin, 21 April 2023. UFRJ 13677, 2 exs.; tributary of Rio do Peixe, 22°52′34.35″ S 45° 56′ 1.52″ W; altitude about 864 m asl; C. Feltrin; 21 April 2023. UFRJ 13678, 1 ex (DNA); tributary of Rio do Peixe 22°,52′34.35″ S 45° 56′ 1.52″ W; altitude about 864 m asl; C. Feltrin; 21 April 2023. UFRJ 13679, 2 exs. (DNA); tributary of Rio do Peixe, 22°52′19″ S 45°55′41″ W, about 910 m asl; C.R.M. Feltrin; 21 April 2023. UFRJ 13680, 1 ex.; upper section of the Rio Santa Bárbara, basin of the Rio do Peixe: 22°51′28″ S 45°54′52″ W, about 1041 m asl; C.R.M. Feltrin, 21 April 2023. UFRJ 13369, 9 exs; Upper Rio do Peixe: 22°55′01″ S 45°57′57″ W; C.R.M. Feltrin, 16 December 2021. UFRJ 13676, 3 exs.; upper section of Rio Santa Bárbara, tributary of Rio do Peixe, 22°50′51″ S 45°54′16″ W, about 1273 m asl; C.R.M. Feltrin, 21 April 2023.

***Trichomycterus mutabilicolor* Costa, 2022**

All from Brazil: Estado de São Paulo: Município de Cunha: upper Rio Paraitinga drainage, Rio Paraíba do Sul basin. UFRJ 12650, H; UFRJ 12649, 1 P; Cachoeira Mato Limpo, Riacho Pedacinho do Céu, a tributary of the Rio Taboão, which is a tributary of the Rio Manso, itself a tributary of the Rio Monjolo, upper Rio Paraitinga drainage, Rio Paraíba do Sul basin, 23°09′25″ S 44°5l′21″ W, altitude about 1335 m asl; B. Mesquita and P.F. Amorim, 25 January 2020. UFRJ 5697, 1P; same locality as holotype; W. J. E. M. Costa, B. B. Costa and C. P. Bove, 8 March 2003. UFRJ 5652, 2P; UFRJ 5696, 1P (C&S); same locality as holotype; W. J. E. M. Costa, B. B. Costa and C. P. Bove, 14 April 2001. UFRJ 5698, 7P; CICCAA 02712, 4P; UFRJ 5699, 3 P (C&S); Toca das Andorinhas, 23°06′08″ S 44°5l′56″ W, altitude about 1200 m asl; W. J. E. M. Costa, B. B. Costa and C. P. Bove, 6 March 2003. UFRJ 5700, 8P; UFRJ 5625, 4P (C&S); same locality and collectors as UFRJ 5698, 17 April 2001.

***Trichomycterus nigroauratus* Barbosa & Costa, 2008**

All material from Brazil, Rio Paraíba do Sul basin. **Estado de São Paulo: Município de São José do Barreiro:** UFRJ 6034, H; UFRJ 5689, 7 P; UFRJ 7585, 6 P; Cachoeira da Usina, Rio do Barreiro, tributary of Rio Paraíba do Sul basin, 22°40′30″ S 44°34′50″ W, about 600 m asl; W.J.E.M Costa, B.B. Costa & C.P. Bove, 19 January 2003. UFRJ 5248, 36 P; UFRJ 5691, 10 P (C&S); same locality as holotype; W.J.E.M Costa, B.B. Costa & C.P. Bove, 25 March 2001. **Município de Piquete:** UFRJ 5692, 7 P; UFRJ 5694, 1 P; UFRJ 5695, 1 P; Ribeirão Piquete, 22°35′17″ S 45°12′39″ W, about 795 m asl; W.J.E.M Costa, B.B. Costa & C.P. Bove; 19 January 2003. UFRJ 705, 3 P; UFRJ 5166, 1 P (C&S); UFRJ 5594, 3 P; UFRJ 4552, 1 P (C&S); tributary of Rio Benfica, 2 km from Piquete; W.J.E.M. Costa & U. Neira, 2 December 1991. UFRJ 662, 4 P; UFRJ 5048, 8 P; UFRJ 5167, 2 P (C&S); same locality as UFRJ 705; W.J.E.M. Costa & M.A. Barbosa, April 1998. UFRJ 10328, 5 exs.; Riacho Tamborete, tributary of Rio Benfica: 22°35′14″ S 45°12′38″ W, about 790 m asl; A. Katz et al.; 19 February 2015. UFRJ 12358, 2 exs.; same locality as UFRJ 10328; B. Mesquita & P. Amorim, 24 July 2019. UFRJ 12360, 1 ex.; Ribeirão Benfica, 22°35′31″ S 45°12′14″ W, about 720 m asl; B. Mesquita and P. Amorim, 24 July 2019. **Município de Pindamonhangaba:** UFRJ 10540, 1 ex: Estrada Jesus Antônio de Miranda, Distrito de Ribeirão Grande: 22°47′10″ S 45°27′18″ W; about 625 m asl; A. Katz et al., 1 June 2015. **Estado do Rio de Janeiro: Município de Itatiaia:** UFRJ 597 2 P; Rio Bonito; W.J.E.M. Costa & C.P. Bove, 10 November 1991. UFRJ 5690, 2 P; Rio Campo Belo, 22°28′32″ S 44°34′44″ W, about 490 m asl; W. Costa et al.; 24 January 2003. UFRJ 4068, 1 P; Rio das Pedras, Três Cachoeiras, Penedo; F. Pupo, 21 November 1996. **Município de Resende:** UFRJ 5154, 1 P (C&S); tributary of Rio Alambari: 22°23′43″ S 44°31′47″ W, about 660 m asl; W. Costa, B.B. Costa & C.P. Bove, 17 June 2000. UFRJ 8465, 1 ex.; Rio Alambari, Serrinha, 22°50′59″ S 44°10′47″ W; about 660 m asl; J. Mattos & J. A. Mattos; 27 July 2010. UFRJ 10490, 3 ex.; tributary of Rio Alambari, Serrinha, 22°35′14″ S 45°12′38″ W, about 790 m asl; J.O. Mattos & M.A. Barbosa; 5 June 2015. UFRJ 12364, 1 ex: Rio Salto, under the bridge on BR-354: 22°25′30″ S 44°44′31″ W, about 980 m asl; B. Mesquita & P. Amorim; 25 June 2019.

***Trichomycterus quintus* Costa, 2020**

All from upper Rio Preto drainage, a tributary of Rio Paraibuna drainage, Rio Paraíba do Sul basin, southeastern Brazil. Estado do Rio de Janeiro: Município de Resende. UFRJ 12601, H; lower Rio Marimbondo, near the confluence with Rio Preto, 22°20′03″ S 44°32′30″ W, about 1000 m asl; W. J. E. M. Costa et al., 25 September 2018. UFRJ 609, 3 mmSL; same locality as holotype; W. J. E. M. Costa & C. P. Bove, 9 November 1991. Estado de Minas Gerais: Município de Bocaina de Minas: UFRJ 12600, 2P; Rio Preto just above Piscina Natural, 22°19′26″ S 44°34′57″ W, about 1120 m asl; W. J. E. M. Costa, 25 October 2019. UFRJ 12597, 1P; same locality and collector as UFRJ 12600; 27 October 2019. UFRJ 12599, 2P; same locality and collector as UFRJ 12600, March 2019. UFRJ 11844, 3P (C&S); same locality as UFRJ 12600; W. J. E. M. Costa & B. Mesquita, 27 December 2017. UFRJ 13920, 2 ex.; same locality as UFRJ 12600; W. Costa et al., 31 July 2023. UFRJ 13783, 3 ex.; same locality as UFRJ 12600; W. Costa et al. 27 July 2023.

## Appendix B

**Table A1:** Terminal taxa, GenBank accession numbers, voucher numbers, and localities for the *CYTB* sequences used in the molecular analyses.

Species	Access Number	Voucher	Locality
*Trichomycterus septemradiatus*	MT470414.1	UFRJ 9888	Conceição da Aparecida, MG
*Trichomycterus paquequerense*	MK144348.1	UFRJ 10001	Lima Duarte, MG
*Trichomycterus santaeritae*	MT470416.1	UFRJ 12408	Rio Preto, RJ
*Trichomycterus mutabilicolor*	OK247577.1	UFRJ 12641	Cunha, SP
*Trichomycterus mutabilicolor*	OK247576.1	UFRJ 12642	Cunha, SP
*Trichomycterus quintus*	OK247575.1	UFRJ 12037.1	Resende, RJ
*Trichomycterus quintus*	OK247574.1	UFRJ 12022	Bocaina de Minas, MG
*Trichomycterus maculosus*	OR995281.1	UFRJ 13675	São José dos Campos, SP
*Trichomycterus maculosus*	MT470417.1	UFRJ12340.1	São José dos Campos, SP
*Trichomycterus nigroauratus*	OK247573.1	UFRJ12357.1	Resende, RJ
*Trichomycterus nigroauratus*	OK247572.1	UFRJ10315	Resende, RJ
*Trichomycterus nigroauratus*	OK247571.1	UFRJ10308	Piquete, SP
*Trichomycterus nigroauratus*	OK247569.1	UFRJ10305.1	São José do Barreiro, SP
*Trichomycterus nigroauratus*	OK247570.1	UFRJ10305.2	São José do Barreiro, SP
*Trichomycterus nigroauratus*	PV472170	UFRJ 12352.1	Piquete, SP
*Trichomycterus rioclarensis*	PV472166	UFRJ 12111.1	Rio Claro, RJ
*Trichomycterus rioclarensis*	PV472167	UFRJ 12111.2	Rio Claro, RJ
*Trichomycterus rioclarensis*	PV472168	UFRJ 13979.1	Rio Claro, RJ
*Trichomycterus rioclarensis*	PV472169	UFRJ 13979.2	Rio Claro, RJ
*Trichomycterus elegans*	PV472171	UFRJ 12470.1	Teresópolis, RJ
*Trichomycterus elegans*	PV472173	UFRJ 13763.2	Três Picos, RJ
*Trichomycterus elegans*	PV472172	UFRJ 13762	C. Frades, RJ

## Appendix C

**Table A2:** Best-fitting partition schemes with their respective numbers of base pairs and the best-suited evolutionary models.

Partition	Base Pairs	Evolutive Model
*CYTB* 1st	360	K80 + I
*CYTB* 2nd	359	HKY
*CYTB* 3rd	359	GTR + G
